# Auditory Responses of Engrailed and Invected-Expressing Johnston’s Organ Neurons in *Drosophila melanogaster*

**DOI:** 10.1371/journal.pone.0071419

**Published:** 2013-08-05

**Authors:** Adeline Pézier, Jonathan M. Blagburn

**Affiliations:** Institute of Neurobiology, University of Puerto Rico, San Juan, Puerto Rico, United States of America; Yale School of Medicine, United States of America

## Abstract

The roles of the transcription factor Engrailed (En), and its paralogue Invected (Inv), in adult *Drosophila* Johnston’s Organ sensory neurons are unknown. We used *en-GAL4* driven CD8-GFP and antibody staining to characterize these neurons in the pedicel (second antennal segment). The majority of En and Inv-expressing Johnston’s Organ neurons (En-JONs) are located in the ventral part of the posterior group of JONs, with only a few in the medial group. Anatomical classification of En-JON axon projections shows they are mainly type A and E, with a few type B. Extracellular recording of sound-evoked potentials (SEPs) from the antennal nerve was used along with Kir2.1 silencing to assess the contribution that En-JONs make to the auditory response to pure-tone sound stimuli. Silencing En-JONs reduces the SEP amplitude at the onset of the stimulus by about half at 100, 200 and 400 Hz, and also reduces the steady-state response to 200 Hz. En-JONs respond to 82 dB and 92 dB sounds but not 98 dB. Despite their asymmetrical distribution in the Johnston’s Organ they respond equally strongly to both directions of movement of the arista. This implies that individual neurons are excited in both directions, a conclusion supported by reanalysis of the morphology of the pedicel-funicular joint. Other methods of silencing the JONs were also used: RNAi against the voltage-gated Na^+^ channel encoded by the *para* gene, expression of attenuated diphtheria toxin, and expression of a modified influenza toxin M2(H37A). Only the latter was found to be more effective than Kir2.1. Three additional JON subsets were characterized using Flylight *GAL4* lines. *inv-GAL4 88B12* and *Gycβ100B-GAL4 12G03* express in different subsets of A group neurons and *CG12484-GAL4 91G04* is expressed in B neurons. All three contribute to the auditory response to 200 Hz tones.

## Introduction

Engrailed (En) is a homeodomain-containing transcription factor found in all bilaterian animals [1], [2], but first identified in *Drosophila melanogaster*, where it plays a crucial part in the patterning of body segments and limbs [3]–[6]. However, the most highly conserved role of En is in neuronal development.

In vertebrates, En is required for cerebellar patterning [7], [8], and formation of the retino-tectal projection [9]–[12]. En regulates the development of spinal cord interneurons [13], [14], and affects the survival of dopaminergic midbrain neurons [13], [15]. In *Drosophila* and grasshopper CNS En controls neuron/glia fate decisions, neuronal identity and axon pathfinding [16]–[18], while in cockroach mechanosensory neurons, we showed that it also controls axon guidance, synaptic target recognition and, as a result, escape behavior [19]–[23].

Despite its well-known role in patterning the *Drosophila* embryo, until recently there were few indications that En played any role in the adult nervous system. Now it is known that subsets of neurons in the peripheral and central nervous system express the *en* gene through adulthood [24], and it has recently been shown that En expression, in combination with that of other transcription factors, is necessary for specifying olfactory sensillum identity and *odorant receptor* (*Or*) gene expression in the third antennal segment [25], [26].

**Figure 1:**
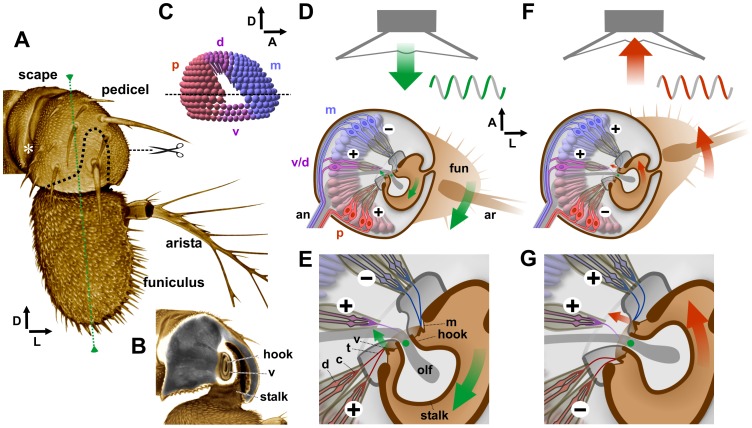
Structure of *Drosophila* Johnston’s Organ (JO). **A**. 3D front view of antenna. Central axis of funiculus shown in green. Cut mark indicates transverse sections shown in D–G; dashed line indicates the funicular stalk. The white asterisk indicates recording electrode insertion site. **B**. The front of the pedicel is removed to show the funicular stalk and hook. Interior contents shown in gray, cuticle is false-colored brown. Between the thick ring of pedicel cuticle and the hook is an elliptical cuticular ring (v). **C**. 3D diagram of the bowl-shaped array of JO neurons (JONs), viewed from medial side, divided into groups by position: posterior (p, red), medial (m, blue), and dorsal (d) and ventral (v) (purple). Line indicates sections in D–G. **D** and **F**. Diagrams of transverse sections through pedicel and JO. **E** and **G**. High magnification views of hinge region. The hollow funicular hook, through which pass the olfactory axons (olf), is anchored in the pedicel by a cuticular membrane (m), the center of which is the axis of rotation (green dot) of the funiculus (fun) and arista (ar). Two JONs are grouped in a single scolopidium, together with scolopale cells (gray ovals). Each neuron terminates in a dendritic cilium (d), inserted into the dendritic cap (c), elongated into a distal thread (t). Threads are attached to cuticular ring (v), coupled to the funicular hook. Posterior and medial groups of scolopidia insert on opposite sides of the cuticular ring; ventrally and dorsally (not shown) are scolopidia that insert on its ventral and dorsal vertices (v/d). **D**, **E**. Posterior and v/d neurons are depolarized (+) by front-to-back movement of the arista; medial group is hyperpolarized (−). **F**, **G**. Medial and v/d JONs are depolarized by back-to-front movement of the arista; posterior group is hyperpolarized. Arrows indicate dorsal (D), lateral (L), and anterior (A) directions.

**Figure 2:**
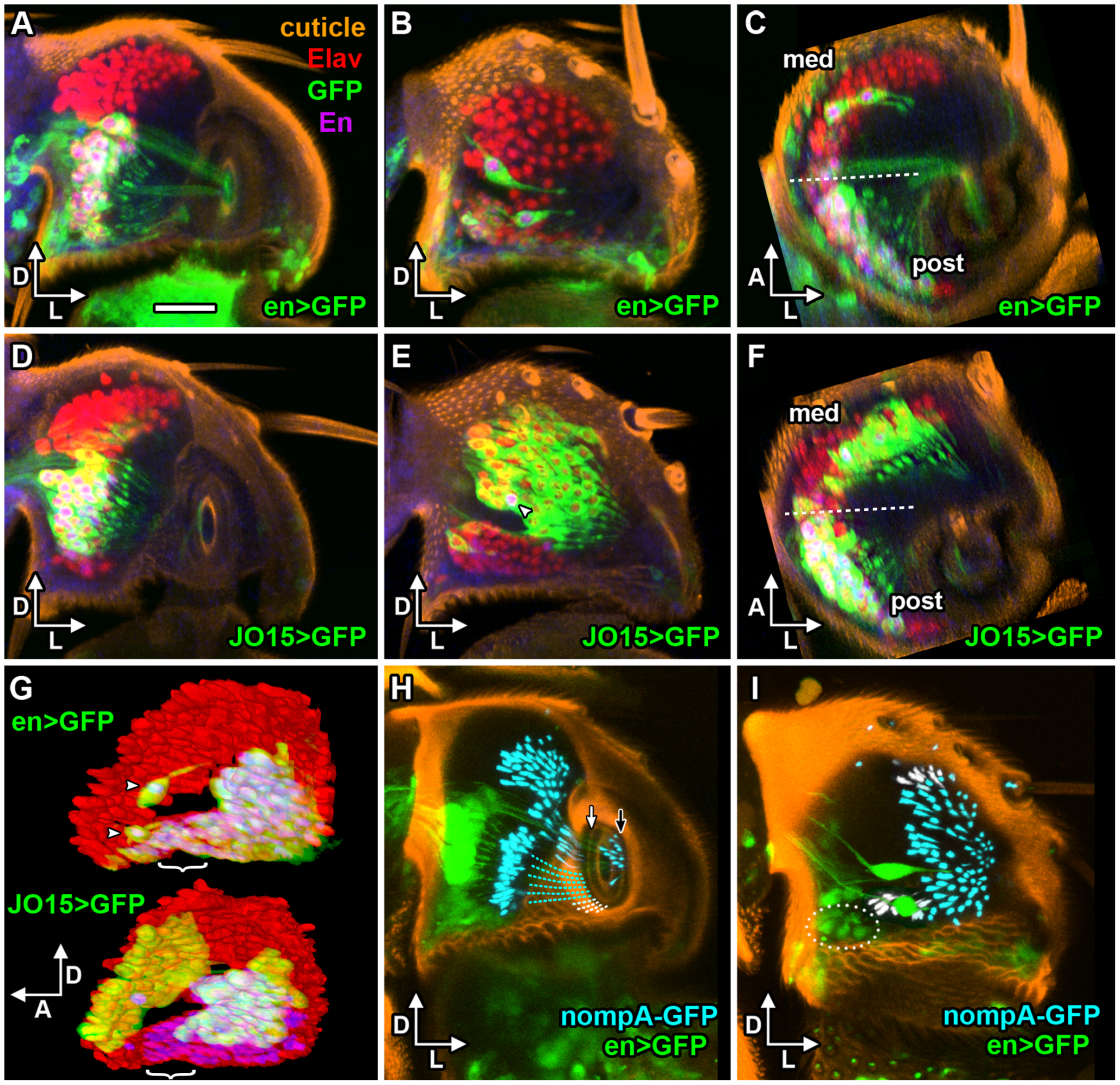
En-expressing neurons in the Johnston’s Organ. **A–G**. mCD8::GFP (green) in the JO of P6 stage left pupal antennae, neuronal nuclei stained with Elav antibody (red), and Engrailed (En) antibody (magenta with Elav staining). **A**. Frontal view of *en*>GFP and En protein in the posterior half of the pedicel, showing En-expressing neurons (En-JONs) only in the ventral half of the posterior group. **B**. Anterior half of same pedicel showing *en*>GFP and En protein only in two large neurons. **C**. Transverse section through pedicel, showing medial and posterior neuronal groups (divided by dashed line). Most En-JONs are in the posterior group. **D**. Frontal view of *JO15*> GFP and En protein in the posterior half of pedicel, showing En expression in many *JO15*-expressing neurons. **E**. Anterior half of the same antenna, showing many *JO15*-expressing neurons, only one of which has En (arrowhead). **F**. Transverse section through pedicel. **G**. 3D views from lateral side of JO, showing ‘open bowl’ arrangement of Elav-positive neurons. En-JONs are present in the posterior and ventral regions of the neuronal array, with only two large neurons in the medial region (arrowheads). *JO15*-expressing neurons are present in both sides of the array. Ventral neurons with En protein (brackets) do not express JO15. **H**, **I**. Frontal views of posterior (H) and anterior (I) halves of pedicel with *en* >2xEGFP (green) and nompA-GFP (cyan or white) to label dendritic caps and threads. Cap threads of most En-JONs (cyan dashes) insert on medial side of cuticular ring (white arrow), only a few ventral En-JONs insert ventrally (white dashes). Anterior caps insert on lateral side of the ring (black arrow). **I**. Dorsally and ventrally inserting threads colored white. Ventral En-JONs encircled with white dots. Dorsal (D), lateral (L), and anterior (A) axes are indicated by arrows. Scale bar: 20 µm.

Engrailed is also expressed in the second antennal segment, or pedicel (Fig. 1 and 2), in a spatially restricted subset of neurons that make up the mechanosensory Johnston’s organ (JO) [24]. The JO is a chordotonal organ containing approximately 200 sensory units called scolopidia, each consisting of 2 or 3 neurons, the eponymous scolopale cell, and ligament and cap cells [27]–[30]. The approximately 480 sensory neurons form a bowl-shaped agglomeration [31], divided anatomically into medial and posterior groups (Fig. 1). Electrophysiological and calcium-imaging studies have shown that some of the JO neurons, or JONs, detect sound (JO-AB neurons), while another subpopulation responds to gravity and wind (JO-CE neurons) [32]–[36]. The JO-A subgroup shows calcium entry in response to sound in a wide range of frequencies ranging from about 100 Hz to 1000 Hz, while the JO-B neurons appear to respond better to lower frequencies [35]. Although many other subsets of JONs can be distinguished with different *GAL4* lines [31], the functional relevance of these is not clear. One possibility, therefore, is that En expression may distinguish a different, overlapping, subset of neurons that perhaps respond to high (or low) frequencies.

Extracellular recordings of sound-evoked potentials from the antennal nerve invariably show oscillations at twice the stimulus frequency [32], [36], [37]. It is not absolutely clear how the mechanics of the insertion of the JO scolopidia at the pedicel-funicular joint gives rise to this frequency doubling. One model postulates that the posterior group of JONs is activated by air moving from the front towards the rear of the animal, and the medial group by air movements in the opposite direction [31], [38]–[40] (Fig. 1), although it was suggested that those JO scolopidia inserted above or below the pivot point (referred to as “v/d” in Fig. 1) would perhaps be activated by both directions [31]. In contrast, a more direct electrophysiological recording method suggests that some individual JO-AB neurons do in fact respond bidirectionally [36], although the anatomical reason for this is not clear.

En-expressing JONs appear to be predominantly located in one anatomical location, the posterior group, and are therefore suited to discriminating between these models – with the former, we would expect them to respond primarily to air moving from front to back. In this study, therefore, we investigate in more detail the anatomical and physiological properties of the En-expressing JONs, and in particular ask whether they respond to sound, and if so at which frequencies, and whether their responses are biased towards front-to-rear movements.

## Materials and Methods

### Flies

*Drosophila melanogaster* were reared on cornmeal media and raised at 25°C. In some cases, to increase *GAL4* activity, flies were transferred to 30°C or, to decrease it, to 18°C [41]. Flies of the following genotypes were obtained from the Bloomington Stock Center: *en-GAL4 e16E* (30564), *JO15-GAL4* on chromosome 3 (6753), *inv-GAL4 88B12* (46851), *Gycβ100B-GAL4 12G03* (48522), *CG12484-GAL4 91G04* (40588), *UAS-mCD8::GFP* (5137), *UAS-Dcr-2, w^1118^; en-GAL4 e16E, UAS-2xEGFP* (25752), *UAS-Dcr-2* (24650), *para-RNAi TRiP Valium 1* (31676), *tubP-GAL80^ts^; TM2/TM6B, Tb^1^* (7108), *nompA-GFP* (42694).

Other lines used were *JO1-GAL4* {Frances Hannan [31]}, *JO15-GAL4* on chromosome 2 (Daniel Eberl), *UAS-Kir2.1, tub-GAL80^ts^* {Kristin Scott [42]}, *tub-GAL80^ts^; UAS-DTI* {Katja Brückner [43]}, *UAS-M2(H37A)-3ME* {Robert Schulz [44]}, *peb-GAL4* {Liqun Luo [45]}. *tub-GAL80^ts/^CyO, Kr-GFP; UAS-M2(H37A)-3ME/TM6B, Tb^1^* flies were constructed in the laboratory.

We used the TARGET system [46] to temporally restrict the expression of some of the drivers. Flies were raised at 18–20°C (permissive temperature for Gal80^ts^). The experimental groups were transferred to 30°C (restrictive temperature for Gal80^ts^) for a limited time as described in the text. Flies maintained at the permissive temperature were used as controls.

### Immunohistochemistry

Adults were used soon after eclosion (approx. 4 h) and at later times (up to 1 week). The animals were anesthetized by cooling, then dissected in phosphate-buffered saline (PBS, 0.1 M). Brains and antennae were immersed in cold fixative (4% paraformaldehyde in 0.1M PBS buffer). For antibody staining of antennae, the heads of pupae at stages P5-P7 [47] were removed and placed in fixative. All tissues were fixed for 30 min, then washed in buffer for approximately 1–2 h. Tissues were first incubated in normal horse serum in PBS +0.3% Triton X 100 (PBST) for 1 h, then in primary antibody, diluted in PBST, for at least 48 h at 4°C. 4D9 (anti-Engrailed and Invected) and nc82 (anti-Bruchpilot) antibodies were obtained from the Developmental Studies Hybridoma Bank (DSHB) and used at a dilution of 1/20. Anti-Engrailed rabbit polyclonal antibody (d-300: Santa Cruz Biotech., CA USA) was used at 1/200. After 4×15 min washes, goat anti-mouse and anti-rabbit antibodies labeled with Alexa-488, Alexa-555, or Cascade Blue (Molecular Probes) were applied at a dilution of 1/400 for 48 h at 4°C, and the tissue was again washed 4 times. The specimens were washed in PBS, then distilled water, then cleared and mounted in Vectashield, then examined with a Zeiss Pascal laser scanning confocal microscope.

Image stacks were imported into ImageJ (Wayne Rasband, NIH), where they were adjusted for optimal contrast. Maximum intensity z-series projections of recombined color stacks were imported into Adobe Photoshop for construction of figures. The 3D viewer plugin was used to make reconstructions of the array of JONs, where cuticular autofluorescence, non-neuronal GFP fluorescence, and dendrites of GFP-labeled neurons were digitally masked from the stack. Other figures were composed using CorelDraw (Corel Corp., Photoshop and Blender (blender.org) software.

### Electrophysiology

Recordings were performed 4–10 days after eclosion. Flies were briefly chilled at 4°C then kept on ice for immobilization before mounting on a slide with dental wax. Sound-evoked potentials (SEPs) were recorded from the antennal nerve using a pair of electrolytically sharpened tungsten electrodes, following previously established methods [32], [37]. One electrode was inserted into the joint between the first and second antennal segments (see Fig. 1A for position). The other electrode was inserted into the head capsule, between the orbital bristles and the eye margin. The signal was amplified x10000 using a differential AC 1700 amplifier (A-M Systems, WA USA) and a Brownlee Precision 210A (Brownlee Precision Co., CA USA) amplifier, bandpass filtered between 10 Hz and 20 kHz, notch-filtered at 60 Hz, digitized with a Digidata 1320A (Molecular Devices LLC, CA USA), and acquired and sampled at 50 kHz with pClamp 8.2 (Molecular Devices). The auditory stimuli consisted of computer-generated pure tones of 200 or 400 ms duration delivered at 1 Hz in trains of 20 stimuli via a MPA-50 40 Watt PA amplifier (Radio Shack) and an Optimus loudspeaker placed 20 cm in front of and facing towards the fly’s head. In order to prevent an abrupt sound onset, which generates a large transient response irrespective of the frequency of the tone, the first and last two cycles of the sine wave were given a gradual onset and offset. The sinusoid frequencies tested were 100, 200, and 400 Hz, and both positive and negative onset waveforms were tested. It was not possible to generate pure tones of lower frequencies using the available equipment. For each frequency, the sound pressure level was adjusted to approximately 90 dB using a RadioShack digital sound meter. Higher (approx. 98 dB) and lower (approx. 82 dB) sound levels were also tested. Equipment to measure the actual sound particle velocities was not available, however, this was not strictly necessary for this study since we do not compare across responses to different frequencies, we only measure the differential effects of silencing neuronal populations within each frequency.

Responses to 20 consecutive stimuli were averaged. The peak to peak amplitude of SEPs of interest was measured using Clampfit (Molecular Devices). Data are presented as mean ± SEM. The normality of the distribution of the data sets was first determined using PAST software (Øyvind Hammer, Oslo University). All statistical tests were then performed using KyPlot (KyensLab Inc). To identify significant differences between means of control vs. experimental groups, normally distributed data were compared using a T-test, whereas non-normally distributed data were compared using a Mann-Whitney test. Significance between experimental groups or between control groups was assessed with a one-way ANOVA, followed by a *post-hoc* Tukey test.

## Results and Discussion

### The Funiculus Rotates about the Insertion of the Funicular Stalk, not its Axis

Before investigating in detail the distribution of the En-expressing neurons, it is important to understand how their positioning relates to the mechanics of the pedicel-funiculus joint. The detailed anatomical study of Göpfert and Robert [38] shows that the funicular stalk is suspended in the pedicel only by a flexible ring of membrane at the end of the “hook” on its antero-medial side (Fig. 1), with no suspensory elements present on its lateral side or at its dorsal tip. We conclude that the end of the funicular hook, and not the axis of the funicular stalk, must therefore represent the axis of rotation of the funiculus. This conclusion is corroborated by laser measurements in that same study [38], which demonstrate that the funiculus rotates symmetrically about its center line when viewed from the anterior of the animal (Fig. 1A), despite the center of the funicular stalk being displaced at least 10 microns laterally from this center line (Fig. 1B). Thus the common depiction of the JO with the funiculus rotating about the center axis of the funicular stalk and the end of the hook moving in a postero-medial or antero-lateral direction (the lock and key model), is not in fact accurate. Instead, it should be shown as rotating about the insertion of the hook (Fig. 1D-G). Attached to the funicular hook at this point is a heavily-sclerotized, presumably stiff, oval ring of cuticle that is V-shaped in cross-section [38], to which are attached the threads that form the distal ends of the dendritic caps of the scolopidia. This ring will move fairly small distances in antero-medial or postero-lateral directions (Fig. 1E, G), i.e. at right angles to the directions previously described. Depending on the mechanical properties of the various constituents of the scolopidia, the threads of the dendritic caps, and the cuticular elements of the pedicel-funicular joint, this anatomical configuration could perhaps still result in alternate stretching of the posterior and medial groups of JON dendrites as per the current model [38]–[40] (illustrated in Fig. 1D-G). However, as the diagram shows, it could clearly also allow for the possibility of bidirectional excitation of all JONs, as originally suggested by Eberl *et al*. [29], [32].

### Engrailed Expression is Mainly in Posterior JO Neurons, in Both Sound- and Gravity/wind-Responsive Classes

The *GAL4* enhancer trap line *en-GAL4 e16E* was used to drive CD8::GFP expression in *en*-expressing JONs [24]. As shown previously, these neurons lie predominantly in the ventral-most half of the posterior group of receptors (Fig. 2A, C), with some also in the ventral group of JONs (Fig. 2G). In contrast, there are only two large JONs in the medial group (Fig. 2B, C). There are approximately 89±6 (N = 5) *en-GAL4*-expressing neurons in total. As shown previously [24], and confirmed here, all such neurons contain some immunoreactivity for the Engrailed and Invected proteins, although there is not necessarily a correlation between the intensity of antibody staining and that of CD8::GFP fluorescence. It is therefore reasonable to conclude that the *e16E* insertion faithfully reports the expression of the *engrailed* gene. In comparison, the *GAL4* line *JO15* [48] labels JO-AB neurons, which probably represent the majority of the sound-responsive JONs [35], and which are divided approximately equally between the medial and posterior groups (Fig. 2D–F). In the posterior group, most *JO15*-labelled AB neurons also express En. There are, however some ventral En-expressing JONs (En-JONs) which are not labeled with *JO15* (Fig. 2G) and probably belong to the C, D or E groups.

It has been suggested [38], [39] that the anatomical division into posterior and medial groups of receptors, each group attached to opposite sides of the funicular hook and being stretched alternately at each phase of the sound wave, would account for the frequency doubling observed in the compound electrical response recorded from the auditory nerve [32]. If this is the case, the position of En-JONs suggests that they should respond primarily to air movements from the front towards the rear of the animal, which would move the arista backwards and thus perhaps stretch the dendrites of these neurons. However, it was recently shown that JO-AB neurons are excited by movement in both directions [36], with the tentative explanation for this being that they could be instead inserted on either the dorsal or ventral sides of the funicular hook. The use of nompA-GFP [49] to label the array of dendritic caps (Fig. 2H, I) suggests that this may not be the case. The majority of the JO-AB neurons (labeled with *JO15*) are located in the posterior and medial groups ([31] and Fig. 2G). The dendritic caps of these groups appear to insert mainly on the elongated sides of the elliptical cuticular ring (Fig. 2H, I), as do the dendritic caps of most of the En-JONs (threads indicated by cyan dashed lines in Fig. 2H). Only a few of the ventral En-JONs (dotted oval in Fig. 2I), probably those which do not express *JO15* (white brackets in Fig. 2G), have caps that seem to be inserted on the ventral vertex of the ring (white caps in Fig. 2I and white dashes in Fig. 2H). Given this, the current model would predict that the asymmetrically-located En-expressing JONs should respond to air movements in a front-to-back direction.

**Figure 3:**
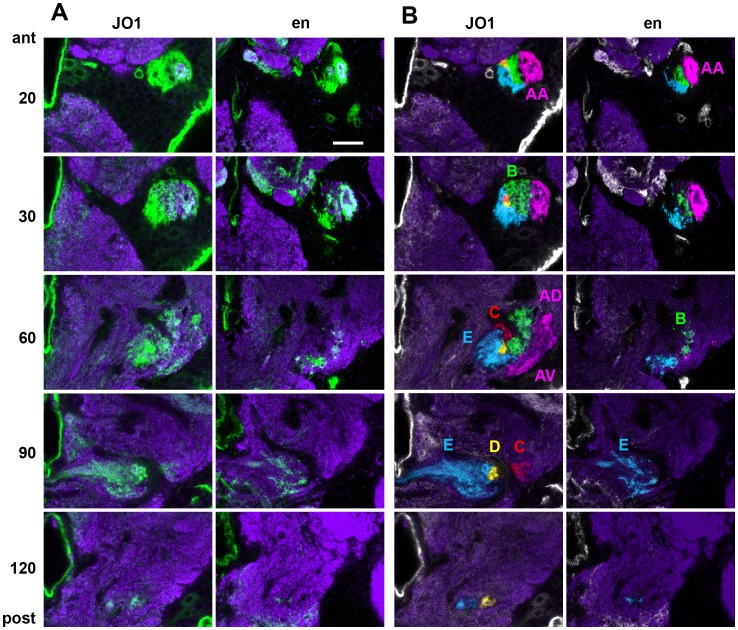
Axonal projections of En-expressing neurons. **A**. mCD8::GFP expression driven by *JO1-GAL4* and *en-GAL4* (green). Background neuropil is stained with nc82 antibody (purple). Vertical columns show representative slices in an anterior – posterior series of confocal slices taken through the brain, in the area of the AMMC and antennal lobe. The numbers indicate the approximate position of the section in microns. **B**. False-colored versions of the GFP images, tinted to show the anatomically defined groups of JO axonal projections as defined by Kamikouchi. Engrailed is expressed in the A group, mostly in the AA subset, in a few B axons, and in some E group axons. Scale bar: 20 µm.

In order to determine the anatomical projections of the En-expressing neurons, and thereby their subgroup identity, we compared *en*-driven GFP to that driven by the *GAL4* enhancer trap line *JO1* (*NP0761*) which expresses in 94% of JONs [31]. Figure 3A shows a side-by-side comparison of confocal slices at different anterior-posterior levels of the *JO1*>GFP and *en*>GFP afferents, with false colors in Figure 3B indicating the afferent groups as defined by Kamikouchi *et al.* [31]. It is clear from this anatomical comparison that En expression is strongly present within the A group, mainly in the anterior (AA) subset, with only a few axons in the ventral (AV) subdivision and none in the dorsal (AD) (Fig. 3B). A small number of B group afferents also express En, but most of the other En-expressing afferents can be classified as the E group (Fig. 3B). No En expression was detected in the easily-identified, posteriorly-projecting D group, or in the less obvious C group. Calcium imaging studies of the JO and its afferents have indicated that the AB neurons respond to soft sound and transient vibrations, while the CE group have some response to sound-like stimuli but also respond strongly to sustained deflections brought about by gentle wind and gravity [34], [35]. Within the CE group, E afferents respond selectively to antero-posterior movement of the arista, while C neurons respond preferentially in the opposite direction [34]. It might therefore be predicted that the *en*-expressing E afferents detect wind and/or gravity, presumably only those stimuli which produce antero-posterior movements of the arista and funiculus; however, these modalities are not the primary objective of this study. Within the AB group of sound-sensitive neurons, B neurons appear to be selective for low frequencies of vibration (approximately 20–200 Hz), while the A neurons detect higher frequencies (from about 100 Hz up to at least 1000 Hz) [35]. Our anatomical results lead to the prediction that En-expressing JO-AB neurons would respond to sound, with the preponderance of A neurons perhaps giving a bias towards higher frequencies. We therefore set out to test this idea using electrophysiology.

### JONs Expressing En Respond to Both High and Low Frequency Sounds, in Both Directions

Our initial approach was to electrically silence the En-expressing JONs and determine which part of the response to sound was affected. For this we used the TARGET system [46], in which ubiquitously-expressed temperature-sensitive Gal80 is used to inhibit Gal4 at low (permissive) temperatures, but can be inactivated at 29–30°C (restrictive temperature) allowing Gal4 to bind to *UAS*. This was coupled with ectopic expression of the human inwardly-rectifying potassium channel Kir2.1, which hyperpolarizes the neurons, reducing the probability of action potentials [50]. The *en-GAL4* driver was employed to target Kir2.1 expression to En-expressing neurons and, to prevent lethality during development, animals were switched to the restrictive temperature only after eclosion. In the experimental group, flies were exposed to the restrictive temperature for Gal80^ts^ (30°C) for two days beforehand, while controls were maintained at the permissive temperature until being cold-anesthetized prior to experimentation.

**Figure 4:**
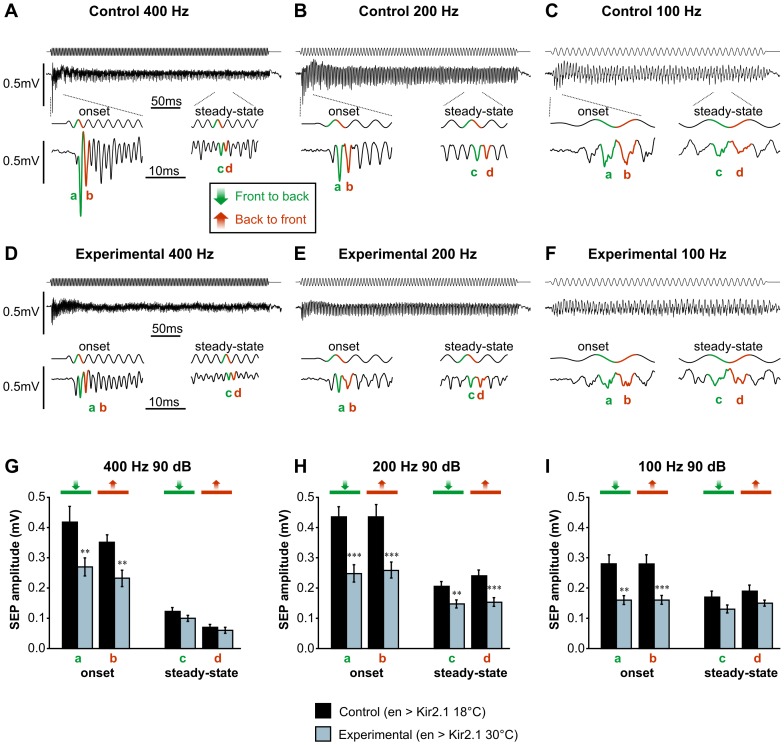
Silencing JONs expressing En causes a global reduction in sound response. Sound-evoked potentials (SEPs) from the antennal nerve were recorded in response to sinusoidal sound wave stimulations in flies whose JONs expressing En were prevented from firing (experimental) or not (control). Firing was blocked by temporarily allowing, using the TARGET system, the expression of the hyperpolarizing potassium channel Kir2.1 under the control of the *en-GAL4* driver in adult flies (genotype: *en-GAL4/+; UAS-Kir2.1,tub-GAL80^ts/^+*; flies were raised at 18-20°C with the experimental group being treated for 2 days at 30°C before recording to allow expression of Kir2.1). **A–C**. Example traces of SEPs in response to 400, 200 and 100 Hz sinusoids at 90 dB in a control fly. Two SEPs are generated per cycle, presumably in response to the movement from front-to-back (green) and back-to-front (red) of the arista. The upper traces are voltage signals sent to the loudspeaker, and hence occur slightly earlier than would actual sound recordings. **D–F**. Example traces of SEPs from an experimental fly recorded as in A showing a conservation of the number of SEPs but a decrease in their amplitude. **G–I**. Amplitude histograms of the SEPs indicated by green and red lines in the example traces in control (N = 15) and experimental (N = 22) flies, showing a significant decrease in amplitude of onset SEPs in flies whose JONs expressing En are silenced, for all three frequencies tested. At 200 Hz (**H**), the steady-state SEPs are also significantly reduced. Significant differences between control vs. experimental means were assessed using T-tests: *** p<0.001, ** p<0.01, * p<0.05.

Sound-evoked potentials (SEPs) were recorded from the antennal nerve in response to sinusoidal sound waves of a range of frequencies (400, 200, and 100 Hz), at three different volumes (approx. 98, 90 and 82 dB). As noted in previous studies [51], at these frequencies the SEPs in response to the first few cycles of the sound wave were significantly larger than the steady-state response (Fig. 4A–C) – these were measured separately (“onset”). In response to these pure-tone stimuli, in control flies, two SEPs were generated per sine cycle (Fig. 4A–C), as first described by Eberl *et al.* [32]. According to the working hypothesis, these peaks should correspond to the alternate activation of the medial and posterior groups during the movement from front-to-back (green) and back-to-front (red) of the arista.

Experimental flies in which Kir2.1 was driven by *en-GAL4* showed the same number of SEPs, but these were of smaller amplitude at all frequencies tested (Fig. 4D–F). For 400 and 100 Hz tones at 90 dB, *en*-driven silencing resulted in significant decreases in the transiently larger response to the sound onset, with no significant effect on the steady-state responses (Fig. 4G and I). This would suggest that *en*-expressing JONs respond only to the onset of the sound at 400 and 100 Hz. However, at 200 Hz, both the response to the sound onset and the steady-state response were significantly reduced, by approximately the same proportion (30–45%), suggesting that, at this frequency, *en*-expressing JONs contribute to both (Fig. 4H). Importantly, at all three frequencies, paired t-tests showed there was no difference between the reduction in amplitude for front-to-back movements versus that for back-to-front movements, suggesting that the *en*-expressing JONs are excited in both directions, thus arguing against the working hypothesis.

The effects of two different volumes were tested at 200 Hz (Table 1). With a louder 200 Hz stimulus (98 dB), there was no significant effect of *en*-JON silencing – this could simply be due to the lower experimental numbers, however, at quieter volumes (82 dB), there was a significant effect with the same N, suggesting that *en*-expressing JONs respond better to quieter sounds rather than louder.

**Table 1:** Effect of different sound volumes on *en*-JON silencing.

	200Hz at 98dB			200Hz at 90dB			200Hz at 82dB	
	control	experimental		Control	experimental		control	experimental
	en>Kir 18°C	en>Kir 30°C		en>Kir 18°C	en>Kir 30°C		en>Kir 18°C	en>Kir 30°C
	(N = 7)	(N = 7)		(N = 15)	(N = 22)		(N = 7)	(N = 7)
**a**	0.45±0.07	0.31±0.05 ns	**a**	0.44±0.03	0.25±0.03 ***	**a**	0.16±0.03	0.08±0.01*
**b**	0.35±0.04	0.27±0.05 ns	**b**	0.44±0.04	0.26±0.03***	**b**	0.19±0.04	0.12±0.02 ns
**c**	0.22±0.03	0.17±0.02 ns	**c**	0.21±0.02	0.15±0.01**	**c**	0.11±0.01	0.08±0.01 ns
**d**	0.21±0.02	0.15±0.02 ns	**d**	0.24±0.02	0.15±0.01***	**d**	0.12±0.01	0.08±0.01*

Mean SEP amplitudes measured from the selected SEPs as designated in Fig. 4 (‘a–b’ for onset and ‘c–d’ for steady-state). Means (mV) ± SEM; N = number of flies tested per condition. Genotypes: *en-Gal4/+;UAS-Kir2.1,tub-Gal80ts/+* (*en*>Kir). T-test was used to assess significant differences between control (flies raised at 18°C) vs. experimental (flies treated for 2 days at 30°C) means.

*** *p*<0.001,

** *p*<0.01,

* *p*<0.05.

As an additional control, we also tested whether exposing the flies to different temperatures had any effect on the responses. We compared the SEP amplitudes of 200 Hz responses of two different crosses raised for two days at 30°C with our Kir 18°C controls and found no significant differences. For example, the first SEP (“a”) in animals of genotype *w^−/^+; JO15-GAL4/+* was 0.50±0.04 mV in amplitude (N = 13), versus 0.43±0.02 mV in Kir controls (N = 63). Animals with a very strong sensory neuron driver, *peb-GAL4*, driving Dicer-2 expression were similarly unaffected: SEP amplitude 0.45±0.06 mV (N = 12).

### Influenza Toxin is More Effective than Kir at Removing the Contribution of *En*-expressing JONs

**Figure 5:**
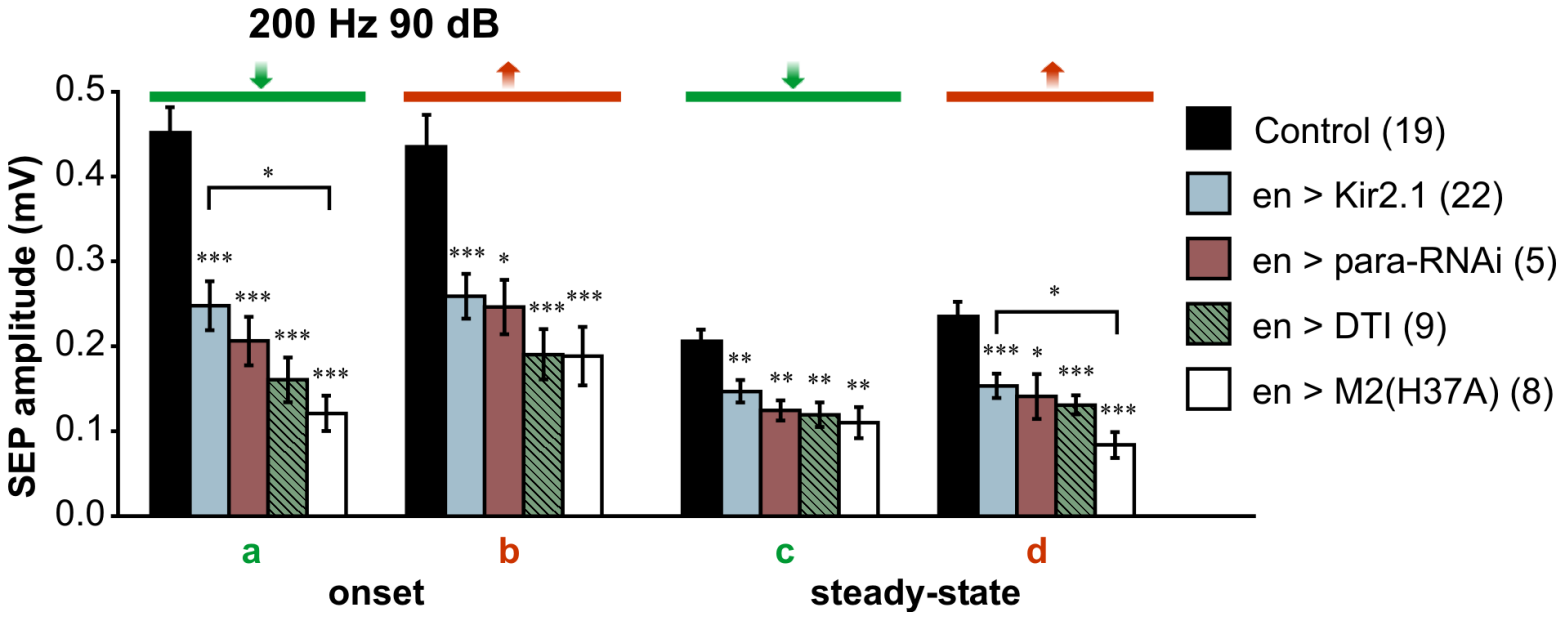
Silencing or poisoning En-expressing JONs affects sound responses similarly. Mean amplitude histograms of the SEPs measured as designated in Fig. 4 for 200 Hz in flies whose JONs expressing En were silenced or poisoned. Silencing was achieved by ectopic expression of the human hyperpolarizing potassium channel Kir2.1 (same bar as figure 4) or by knock-down of the *Drosophila* Na^+^ channel alpha subunit (*para-RNAi*). Poisoning was achieved by expression of the attenuated diphtheria toxin DTI or the modified influenza toxin M2(H37A). Silencing or poisoning led to a reduction of all SEPs measured. Genotypes: *en-GAL4/+; UAS-Kir2.1,tub-GAL80^ts/^+* or *en-GAL4/tub-GAL80^ts^; UAS-M2(H37A)-3ME/+* raised at 18-20°C (control); *en-GAL4/+; UAS-Kir2.1,tub-GAL80^ts/^+* flies treated at 30°C (*en*>Kir2.1), *UAS-Dcr2, w^1118/^+; en-GAL4/+; UAS-para-RNAi/UAS-2xEGFP* (*en* >para-RNAi), *en-GAL4/tub-Gal80^ts^; UAS-DTI/+* (*en*>DTI), *en-GAL4/tub-GAL80^ts^; UAS-M2(H37A)-3ME/+* (*en*>M2(H37A)). Numbers in parenthesis indicate the number of flies tested for each experimental condition. Significant difference between means of each experimental group vs. control was assessed using a T-test. Significance between experimental groups was assessed with a one-way ANOVA followed by a post-hoc Tukey test. *** *p*<0.001, ** *p*<0.01, * *p*<0.05.

At some point during these experiments, the question arose as to whether simply hyperpolarizing the JONs by expressing Kir2.1 would be enough to prevent them from firing action potentials. We therefore tried three other methods of silencing or poisoning the neurons, and tested their effects using pure-tone stimuli at 90 dB (Fig. 5). The first of these strategies was to use RNA interference against the *Drosophila* voltage-gated Na^+^ channel, encoded by the *para* gene. This has been shown to completely inhibit the spontaneous firing of JONs [36]. *UAS-para-RNAi* (TRiP, Valium 1) was driven by *en-GAL4* in a line containing *UAS-Dcr-2*, with lethality during development being prevented by reducing Gal4 expression by raising the larvae and pupae at 18°C. In five experimental animals this proved no more effective than Kir2.1 in reducing the SEPs (Fig. 5), suggesting that Kir2.1 does indeed inhibit the production of Na^+^-dependent action potentials. It should be noted however, that this is not the same RNAi construct as used in the other study [36], and its efficacy may be different. The second strategy was to use *tub-GAL80^ts^* to control the expression of an attenuated mutant version of diphtheria toxin A-chain, which inhibits protein synthesis resulting in neuronal death [52]. This again was not significantly more effective than Kir2.1, however, it can be relatively slow-acting, particularly in the adult [53]. We did however observe that it does eliminate almost all *en*-driven GFP fluorescence (not shown). Finally, we used a modified version of the influenza toxin M2(H37A), which forms a constitutively active non-specific cation channel, to silence and perhaps kill the neurons [54] [55]. This modified toxin has been shown to be equally as effective as ectopic expression of the pro-apoptotic gene *rpr* in ablating *Drosophila* cells, particularly in post-embryonic stages [44]. We found this to be particularly effective in reducing the contribution of *en*-expressing JONs, with a reduction of 30–50% in the SEPs, in some cases up to twice the reduction brought about by Kir2.1 expression (Fig. 5). This suggests that perhaps Kir hyperpolarization is not sufficient to fully silence all *en*-expressing JONs, perhaps particularly those with weaker Gal4 expression; with the additional possibility existing that some JONs may have non-Na^+^-dependent action potentials.

It should be noted that, as with Kir2.1, all these silencing/poisoning methods showed similar degrees of knockdown of SEPs with regard to front-to-back versus back-to-front movements, and to both high and low frequency sound. Taken together with the asymmetrical location of the En-expressing JONs, this means that individual neurons are able to respond to movement in either direction, contrary to the current model [38]–[40]. This bidirectionality does not appear to be due to insertion of the threads of the dendritic caps on the dorsal and ventral vertices of the funicular hook as previously suggested [36]; instead, our revised interpretation of the morphology of the pedicel-funicular joint suggests a way in which stretch-activation of the dendrite of a neuron could take place in both directions (Fig. 1D, F).

### Anatomical Comparison of Different JON Subgroups

The *JO15* driver [48] is known to be expressed in approximately 145 of the JONs (Fig. 2D–F), which are categorized in the A and B groups based on the anatomy of their axonal projections [31]. In calcium imaging studies [34], [35], these neurons were shown to account for most of the response to vibrating deflections of the arista and to quiet, near field sound (the pulse part of the courtship song). We therefore wanted to compare how silencing of this JON subgroup with Kir or M2(H37A) affected the SEPs recorded from the antennal nerve, and compare this to *en*-*GAL4* driven silencing.

In addition, we also identified three more *GAL4* lines, generated by the Flylight Project [56], that express in more restricted subgroups of JONs: GMR 88B12, a fragment of the *invected* enhancer region (referred to here as “*inv 88B12”*); GMR 12G03, from the enhancer of *Gycβ100B* (“*Gycβ100B 12G03”*); and GMR 91G04, from the enhancer of *CG12484* (“*CG12484 91G04”*).

**Figure 6:**
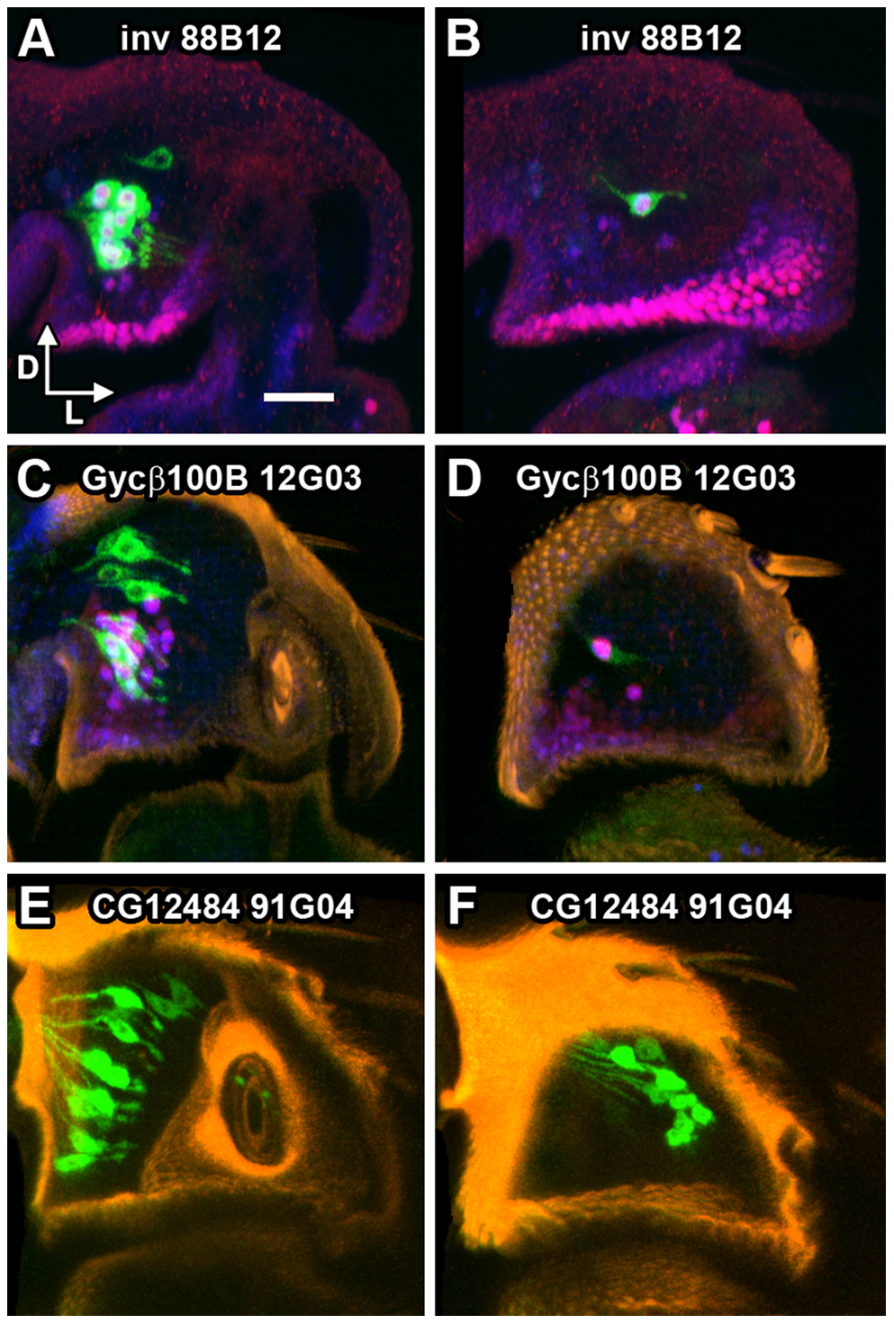
Subsets of JONs expressing GMR GAL4 drivers. mCD8::GFP expression driven by different Flylight *GAL4* drivers (green). **A, B**. 30 h APF pupal pedicel, showing *inv 88B12*> GFP, along with antibodies against En protein (red) and both En and Inv (blue). **A**. Posterior half, with cluster of large En+ neurons. **B**. Anterior half, with a single large En+ neuron. **C, D**. 36 h APF pupal pedicel, showing *Gycβ100B 12G03*> GFP, along with antibodies against En protein (red) and both En and Inv (blue), and cuticular autofluorescence false-colored orange. **C**. Posterior half, with several dorsal and ventral neurons, some of the latter are En+. **D**. Anterior half, with weak expression in a single large En+ neuron. **E, F**. 72 h APF pupal pedicel, showing *CG12484 91G04*> GFP, along with cuticular autofluorescence false-colored orange. **E**. Posterior half, with many dorsal and ventral neurons. **F**. Anterior half, with several neurons. Dorsal (D), lateral (L), and anterior (A) axes are indicated by arrows. Scale bar: 20 µm.

**Figure 7:**
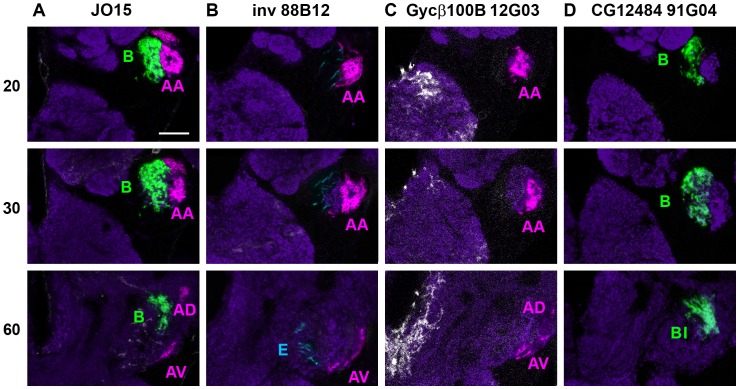
Axonal projections of GMR GAL4 drivers compared to JO15. mCD8::GFP expression driven by *JO15-GAL4* and different Flylight *GAL4* drivers (false-colored to show the anatomically defined groups of JO axonal projections), in anterior – posterior series of confocal slices taken through the brain, in the area of the AMMC and antennal lobe. Background neuropil is stained with nc82 antibody (dark purple). The numbers indicate the approximate position of the section in microns. **A**. *JO-15* drives GFP in the A and B groups of axons, except BI. **B**. *inv 88B12* drives GFP mainly in the A group of axons. **C**. *Gycβ100B 12G03* drives GFP in the A group of axons. **D**. *CG12484 91G04* drives GFP in the B group of axons, including BI. Scale bar: 20 µm.

The *GAL4* driver *inv 88B12* is strongly expressed in a subset of 12–15 large JONs, located mainly in the ventral part of the posterior group (Fig. 6A), with a single neuron in the medial group (Fig. 6B). The majority of these neurons contain both Engrailed and Invected proteins. Strong Gal4 expression is present from approximately 30 h APF (Fig. 6A, B) and perhaps earlier. The axons of this group mainly project in the AA cluster, with some fainter axons in the AV projection and a small number of faintly stained axons in the E group (Fig. 7B).

The *GAL4* driver *Gycβ100B 12G03* has moderate expression in a subset of approximately 10–12 posterior neurons in the JO, the ventral ones being En and Inv-positive, the dorsal ones not (Fig. 6C). There is only weak expression of this driver in one of the medial En-positive neurons (Fig. 6D). Expression begins to appear in scattered JONs at about 30–36 h APF but is not fully present until about 45 h APF, after cuticle apolysis (Fig. 6C, D). Axons expressing *Gycβ100B 12G03* are also restricted to the A projection, with fewer in the AA subgroup than *inv 88B12*, but more in the AV and AD groups (Fig. 7C).

The *GAL4* driver *CG12484 91G04* does not express in JONs until later in pupal development than the others, when antibody penetration into the pedicel is severely restricted. There are approximately 25–35 neurons in both dorsal and ventral portions of the posterior group (Fig. 6E) and 20–30 in the medial group (Fig. 6F). In comparison, the previously-described *JO15* and *en-GAL4* drive expression in neurons mainly in the ventral part of the posterior group, while the medial group has many *JO15*-expressing neurons and only two that express *en* (Fig. 2A-F). Axons expressing *CG12484 91G04* appear to be almost exclusively in the B projection region; although there are fewer than with *JO15*, this driver does however label axons in the BI projection, which *JO15* does not (Fig. 7D).

### Contributions of the Different JON Subgroups to the Antennal Nerve Response

**Figure 8:**
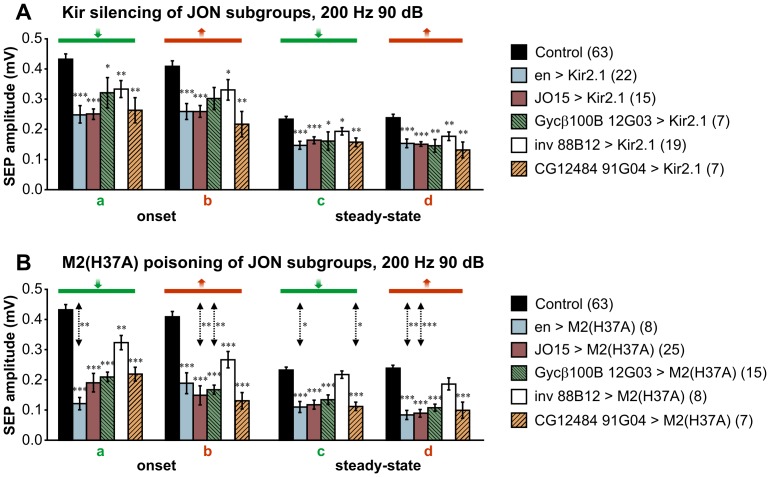
Effects on the sound response of silencing or poisoning different JON groups. Mean amplitude histograms of SEPs in response to 200 Hz at 90 dB, comparing different *GAL4* drivers expressing in different subsets of JONs. **A**. Silencing with Kir. Genotypes: *en-GAL4/+; UAS-Kir2.1,tub-GAL80^ts/^+*, *JO15-GAL4/+;UAS-Kir2.1,tub-GAL80^ts/^+* and *JO15-GAL4/UAS-Kir2.1,tub-GAL80^ts^*, *Gycβ100B-GAL4 12G03/UAS-Kir2.1,tub-GAL80^ts^*, *inv-GAL4 88B12/UAS-Kir2.1,tub-GAL80^ts^*, *CG12484-GAL4 91G04/UAS-Kir2.1,tub-GAL80^ts^* **B**. Poisoning with M2(H37A). Genotypes: *en-GAL4/tub-GAL80^ts^; UAS-M2(H37A)-3ME/+*, *JO15-GAL4/tub-GAL80^ts^; UAS-M2(H37A)-3ME/+* and *tub-GAL80^ts^/+; JO15-GAL4/UAS-M2(H37A)-3ME*, *tub-GAL80^ts^/+; Gycβ100B-GAL4 12G03/UAS-M2(H37A)-3ME*, *tub-GAL80^ts^/+; inv-GAL4 88B12/UAS-M2(H37A)-3ME*, *tub-GAL80^ts^/+; CG12484-GAL4 91G04/UAS-M2(H37A)-3ME*. The control group is a combination of the different genotypes from flies kept at 18°C. Numbers in parenthesis indicate the number of flies tested for each experimental condition. T-tests or Mann-Whitney tests were used to assess the significant differences between control vs. experimental means and Kir vs. M2(H37A) for each driver (vertical double-headed arrows). *** *p*<0.001, ** *p*<0.01, * *p*<0.05.

In order to determine the relative contributions made by the five different JON subgroups (*JO15, en, inv 88B12*, *Gycβ100B 12G03, and CG12484 91G04*) to the antennal nerve response to sound, we employed Kir silencing (Fig. 8A) or M2(H37A) poisoning (Fig. 8B), see also Table 2. With Kir silencing, all JON subgroups showed significant reductions in the amplitude of onset or steady-state SEPs (Fig. 8A). No significant differences between the groups were apparent at 200 Hz.

**Table 2:** Contribution of different JON subgroups to sound-evoked potentials.

Kir2.1 silencing of JON subgroups						
control		en	JO15	Gycβ100B 12G03	inv 88B12	CG12484 91G04
(N = 63)		(N = 22)	(N = 15)	(N = 7)	(N = 19)	(N = 7)
**400Hz at 90dB**						
**a**	0.45±0.02	0.27±0.03***	0.24±0.02***	0.32±0.06*	0.32±0.03***	0.30±0.04*
**b**	0.39±0.02	0.19±0.02***	0.15±0.01***	0.31±0.05 ns	0.25±0.03***	0.31±0.04 ns
**c**	0.14±0.01	0.10±0.01***	0.12±0.01 ns	0.08±0.02*	0.08±0.01***	0.10±0.01 ns
**d**	0.09±0.01	0.03±0.01***	0.07±0.01 ns	0.05±0.01*	0.06±0.01*	0.09±0.02 ns
**200Hz at 90dB**						
**a**	0.43±0.02	0.25±0.03***	0.25±0.02***	0.32±0.05*	0.33±0.03**	0.26±0.04**
**b**	0.41±0.02	0.26±0.03***	0.26±0.02***	0.30±0.04 ns	0.33±0.03*	0.22±0.04**
**c**	0.23±0.01	0.15±0.01***	0.16±0.01***	0.16±0.03*	0.19±0.01*	0.16±0.01**
**d**	0.24±0.01	0.15±0.01***	0.15±0.01***	0.15±0.02**	0.18±0.01**	0.13±0.03**
**100Hz at 90dB**						
**a**	0.27±0.01	0.16±0.14***	0.20±0.02**	0.23±0.03 ns	0.22±0.02*	0.22±0.03 ns
**b**	0.26±0.01	0.16±0.15***	0.16±0.02***	0.19±0.02**	0.22±0.02*	0.14±0.03**
**c**	0.19±0.01	0.13±0.13***	0.16±0.02 ns	0.16±0.02 ns	0.16±0.01*	0.16±0.03 ns
**d**	0.18±0.01	0.14±0.09**	0.13±0.01**	0.15±0.01*	0.15±0.01**	0.14±0.03 ns
**M2(H37A) poisoning of JON subgroups**						
	**control**	**en**	**JO15**	**Gycβ100B 12G03**	**inv 88B12**	**CG12484 91G04**
	(N = 63)	(N = 8)	(N = 25)	(N = 15)	(N = 8)	(N = 7)
**400Hz at 90dB**						
**a**	0.45±0.02	0.19±0.03***	0.23±0.03***	0.19±0.02***	0.42±0.04 ns	0.20±0.03***
**b**	0.39±0.02	0.07±0.02***	0.17±0.03***	0.15±0.02***	0.37±0.03 ns	0.18±0.02***
**c**	0.14±0.01	0.08±0.01***	0.07±0.01***	0.08±0.01***	0.14±0.01 ns	0.07±0.01***
**d**	0.24±0.01	0.01±0.00***	0.04±0.01***	0.02±0.01***	0.10±0.01 ns	0.04±0.01***
**200Hz at 90dB**						
**a**	0.43±0.02	0.12±0.02***	0.19±0.03***	0.21±0.02***	0.32±0.02**	0.22±0.02***
**b**	0.41±0.02	0.19±0.03***	0.15±0.03***	0.17±0.02***	0.27±0.03***	0.13±0.03***
**c**	0.23±0.01	0.11±0.02***	0.12±0.01***	0.13±0.02***	0.22±0.01 ns	0.11±0.01***
**d**	0.24±0.01	0.08±0.02***	0.09±0.01***	0.11±0.01***	0.19±0.02 ns	0.10±0.03***
**100Hz at 90dB**						
**a**	0.27±0.01	0.09±0.02***	0.13±0.02***	0.16±0.02***	0.23±0.02*	0.15±0.02***
**b**	0.26±0.01	0.09±0.02***	0.10±0.02***	0.14±0.01***	0.17±0.02***	0.15±0.02***
**c**	0.19±0.01	0.10±0.02***	0.11±0.01***	0.12±0.01***	0.19±0.01 ns	0.10±0.01***
**d**	0.18±0.01	0.10±0.02***	0.08±0.01***	0.11±0.01***	0.14±0.01**	0.10±0.01***
						

Mean SEP amplitudes measured from the selected SEPs as designated in Fig. 4 (‘a–b’ for onset and ‘c–d’ for steady-state). Means (mV) ± SEM; N = number of flies tested per condition. Genotypes as in legend of Fig. 8. T-test or Mann-Whitney test were used to assess significant differences between control group (flies raised at 18°C) vs. each experimental group (flies treated at 30°C).

*** *p*<0.001,

** *p*<0.01,

* *p*<0.05.

M2(H37A) toxin was again, in several cases, significantly more effective than Kir (Fig. 8B). The silencing effect of the toxin was not significantly different for the various subgroups, except for *inv 88B12*, which was less effective at reducing the SEPs compared to *en-GAL4*, with no significant effect of *inv*>M2(H37A) on the steady-state SEPs (Fig. 8B). This could be due to the relatively small number of neurons labeled by this line. However, a comparatively weaker driver expressing in a slightly different but equally small subset of group A JONs, *Gycβ100B 12G03*, did prove effective, indicating that the loss of the action potentials of less than 20 neurons can indeed make a significant difference to the amplitude of the overall SEP. A more likely explanation is that, in order to avoid lethality, *inv 88B12*> M2(H37A) animals had to be transferred to 30°C after eclosion rather than in late pupal stages as for the other Flylight lines. Exactly why toxin expression in these particular *inv 88B12*-expressing neurons should prove lethal is not clear, unless there is some as yet unidentified expression elsewhere in this line.

We found that the B group of JONs, as labeled by the driver *CG12484 91G04*, also makes a significant contribution to the SEP at 200 Hz, whether assessed using Kir or M2(H37A). However, a previous calcium imaging study suggested that this group (albeit labeled with a different driver, *JO2*) does not respond to large arista deflections at this frequency [35], yet the same neurons were shown to be required for the chaining response to courtship song [35]. In pilot experiments, we found that *JO2* driving Kir2.1 had absolutely no silencing effect on SEPs, even though the animals were moribund after two days at 30°C, indicating expression elsewhere.

En-expressing JONs, comprising anatomical subgroups A, (B), and E, seem to contribute as much to the SEP as do the *JO15*-labeled JONs (subgroups A and B) that have been suggested to represent most of the sound-responsive neurons [34], [35]. In our hands, silencing AB JONs with *JO15* did not completely eliminate the SEP, as might have been expected if these neurons are exclusively responsible for the response to sound. We did find however that while *JO15* on chromosome 3 tended to be variable in its effects, sometimes giving silencing and sometimes not, when it was located on chromosome 2 it was much more reliably effective. The results shown in Figure 8 are combined from animals with the driver at both locations, and so may represent an underestimate of the contribution of JO-AB neurons.

Overall, notwithstanding the inherent difficulties in comparing the effects of different drivers with different levels of expression, it seems clear that En-expressing JONs play an important role in sound detection, as do the three different subgroups of neurons labeled by the Flylight *GAL4* lines. Questions remain about the role of Engrailed and its paralogue – they clearly make up part of the combinatorial system of transcription factors that determine Johnston’s Organ neuronal subtype identity, but we have shown that they appear not to be exclusively expressed in neurons that respond to one modality, such as sound or gravity, nor to movement in a particular direction, nor to a limited frequency range. It is possible that En may be involved in determining other neuronal properties, such as connectivity to interneurons, and we intend to investigate this possibility in the future.
